# Impact of house dust mite-driven asthma on children’s school performance and activity

**DOI:** 10.1007/s00431-021-04346-y

**Published:** 2021-12-21

**Authors:** Catalina Gómez, Judit Barrena, Vanesa García-Paz, Ana M. Plaza, Paula Crespo, José A. Bejarano, Ana B. Rodríguez, Laia Ferré, Lidia Farrarons, Marta Viñas, Carla Torán-Barona, Andrea Pereiro, José L. Justicia, Santiago Nevot

**Affiliations:** 1 Althaia Xarxa Assistencial I Universitària de Manresa, Manresa, Spain; 2 Consorci Sanitari de Terrassa, Terrassa, Spain; 3 Hospital Quirón, Coruña, Spain; 4 Hospital Sant Joan de Déu, Esplugues de Llobregat, Spain; 5 Hospital Virgen del Rocío, Sevilla, Spain; 6 Allergy Therapeutics Ibérica, Avda. Barcelona, 115, 08970 Sant Joan Despí, Barcelona, Spain

**Keywords:** School impairment, Absenteeism, Work impairment, Subcutaneous allergen immunotherapy, Microcrystalline tyrosine, Allergic asthma

## Abstract

Evidence regarding asthma’s impact on children’s daily lives is limited. This prospective and cross-sectional, observational, multicenter study assessed school/work and activity impairment in children and adolescents with allergic asthma and their caregivers and allergen immunotherapy (AIT) effects. Included patients were schooled children and adolescents (5 to 17 years) with allergic asthma due to house dust mites (HDM)*.* Impairment of school/work (i.e., absenteeism and presenteeism) and activity was measured in patients and their caregivers using the Work Productivity Impairment Questionnaire plus Classroom Impairment Questions: Allergy Specific (WPAI + CIQ:AS). HDM allergic patients with school impairment received subcutaneous AIT with a MicroCrystalline Tyrosine-associated allergoid. WPAI + CIQ:AS and effectiveness variables were compared between baseline and 1-year post-AIT. Of the 113 patients included, 59 (52.2%) and 51 (45.1%) showed school and activity impairment, respectively, missing a mean (SD) of 37.6 (24.4) % and 42.6 (25.6) % of school and activity time, respectively. Twenty-six (23%) caregivers reported activity impairment and, of the 79 (69.9%) employed, 30 (38%) reported work impairment. Of the 65 patients with school/activities impairment, 41 (63.1%) received AIT, of which 21 (51.2%) completed 1 year of treatment. Effectiveness variables and WPAI + CIQ:AS significantly improved: Mean (SD) school impairment decreased from 39.7 (26.7) to 2.1 (7.1) % (*p* < 0.001) and activity impairment from 46.2 (34.6) to 1.4 (3.6) % (*p* < 0.001).

*Conclusion:* Allergic asthma due to HDMs results in school/work and activity impairment in children and adolescents and their caregivers. One year of AIT provided clinical benefits and reduced school and activity impairment.

**Table:** 

**What is Known:** *• Allergic asthma impairs children’s school performance and daily activities.* *• Allergen immunotherapy modifies allergic disease course and ameliorates its symptoms*.	
**What is New:** *• Asthma symptoms due to allergy to house dust mites impair children’s school attendance and productivity and daily activity and their caregivers’ work performance and daily lives.* *• Allergen immunotherapy with a house dust mite MicroCrystalline Tyrosine (MCT)-associated allergoid seems to provide clinical benefits, associated with decreased school and activity impairment, supporting it as an effective treatment option.*

**Supplementary Information:**

The online version contains supplementary material available at 10.1007/s00431-021-04346-y.

## Introduction

Allergic diseases, including asthma, rhinitis, and atopic dermatitis, impair multiple aspects of patients’ daily functioning, causing a serious burden and impaired quality of life [1]. In children, asthma is one of the most common chronic diseases, with an estimated global symptom prevalence of 11.6 to 13.7%, and is frequently associated with allergic rhinitis [2]. Among the most frequent allergens associated with allergic asthma, those from house dust mites (HDMs) are common in indoor environments (i.e., at home) and rank second after pollens, constituting the main cause of perennial allergic asthma in Spain [3, 4]. Despite the increased awareness of asthma in children and availability of effective treatment, asthma control is still suboptimal, affecting children’s daily lives and, consequently, their quality of life (QoL) and that of their caregivers [5].

Among the activities impaired by asthma, school attendance is frequently hampered due to asthma exacerbations, lack of asthma control, and routine medical visits, resulting in school absenteeism [6, 7]. In addition, the symptoms’ burden and lack of sleep due to night wheezing impair children’s at-school productivity (presenteeism), further impacting their overall school performance [8]. Moreover, children’s asthma generates indirect costs, including, in addition to those associated with loss of leisure time, the ones related to their caregivers’ activity and work impairment, which have been reported to be higher than direct costs [9]. Among the factors influencing school performance, previous studies have shown that good asthma control decreases school absenteeism and productivity, underscoring a role for regular asthma medication and inhaled corticosteroids [6, 10–12]. In this context, AIT, the only treatment able to modify the course of the disease and ameliorate its symptoms, may decrease children’s school and activity impairments and, consequently, those of their caregivers [13]. Among the different commercially available HDM-specific immunotherapies, Acarovac Plus® has shown a favorable effectiveness profile in previous observational studies [14–17].

Despite the previously appreciated impact of asthma on school performance and activities and, consequently, quality of life of children and adolescents, studies are insufficient and mostly focused on absenteeism [18–23]. Furthermore, to our knowledge, school and activity impairment specifically due to the most common perennial allergen, HDMs, remains unassessed. In this observational, prospective study, we assessed the impact of allergic disease on school performance and daily activities of children and adolescents with allergic asthma caused by HDMs, as well as on work and activities of their caregivers, and the effects of AIT after 1 year of treatment.

## Methods

### Study design and population

This was an observational, prospective and cross-sectional, multicenter study including children and adolescents with allergic asthma, with and without allergic rhinitis, and sensitized to *Dermatophagoides sp*. Schooled patients aged 5 to 17 years who attended visits in five Spanish allergy centers during two school years (October 2017–June 2018 and October 2018–June 2019) were consecutively included in the study. Patients had to be able to understand and complete, independently or with their accompanying adults’ assistance, the administered questionnaire. Patients with concomitant diseases or circumstances potentially interfering with school attendance and patients participating or enrolled to participate in any other clinical study were excluded. According to the investigators’ criteria, based on the routine clinical practice, patients with loss of school time and school impairment were prescribed AIT with Acarovac Plus® (Allergy Therapeutics, Worthing, UK). This AIT is a purified allergen extract of mites—50% *Dermatophagoides pteronyssinus*/50% *Dermatophagoides farinae*—modified with glutaraldehyde and associated to MicroCrystalline Tyrosine [MCT], in an injectable suspension for subcutaneous administration.

Data from patients and their caregivers were collected during a baseline visit (V1) and additionally, for patients who were prescribed Acarovac Plus®, during a final visit (FV) 1 year after V1 (scheduled to match routine visits). Assessments were performed at V1 and FV. All legal representatives of patients signed a written informed consent before any information was recorded. The study was conducted according to the Helsinki Declaration and the local Personal Data Protection Law (LOPD 15/1999); the study protocol was approved by the Ethics Committee of “Fundació Unió Catalana Hospitals.”

### Endpoints, variables, and assessments

The primary endpoint of this study was the impact of allergic disease on school attendance and classroom performance in children and adolescents with asthma caused by *Dermatophagoides spp*. Secondary endpoints were the impact of allergic disease on the activity of children and adolescents with allergic asthma and on work performance and daily activities of their caregivers, and the effects of AIT in children’s school and activity impairment due to allergic disease. The main variable was the impact on school impairment, measured as missed school time (i.e., absenteeism) and loss of classroom productivity (i.e., presenteeism) using the validated Spanish version of the Work Productivity and Activity Impairment Questionnaire plus Classroom Impairment Questions: Allergy Specific (WPAI + CIQ:AS), a patient-reported questionnaire validated in asthmatic patients [24–26]. The questionnaire was administered to patients during V1 and FV and to caregivers during V1 and included nine items assessing the time of work, school, and activities missed during the previous 7 days due to allergic disease. Specifically, the WPAI + CIQ:AS measures school and work absenteeism and presenteeism, overall school and work impairment (absenteeism plus presenteeism), and activity impairment [24]. Outcomes were calculated as the number of patients/caregivers with impairment and impairment percentages, with higher values indicating greater impairment and reduced productivity. Additional variables were essential demographic characteristics of patients and those of caregivers, such as relationship with the patient and work status. Clinical and treatment variables of patients included duration of allergic disease, sensitization to aeroallergens, other allergic diseases, number of visits to the healthcare center during the previous 12 months, use of medication to treat asthma, asthma severity classification, and asthma control. Asthma severity classification and control were graded according to the “Guía Española para el Manejo del Asma v4.0” (GEMA), the Spanish version of the Global Initiative for Asthma (GINA) classification, following, for asthma control, the classification for children [27, 28].

### Statistical analysis

Categorical variables were described as frequencies and percentages, and quantitative variables as the mean and standard deviation (SD). Categorical variables were compared using the chi-square test and the Fisher’s test. Correspondingly, quantitative variables were compared using the Student’s *T*-test and their non-parametric counterparts Mann–Whitney and Kruskal–Wallis tests. The significance threshold for all bivariate analyses was set at a two-sided *α* = 0.05. All analyses were performed using the statistical package support SPSS.

Using the “Estimation of a proportion” method, and assuming a 22.5% loss of activities due to bronchial asthma and a 10% of data missing, a sample size of 210 patients was deemed necessary to calculate the primary endpoint (i.e., the impact of allergic disease in school attendance and performance) with a ± 6% precision and a 95% CI [25]. Additionally, assuming a reduction of school performance due to moderate-severe allergic rhinitis of 40% and a 10% of data missing a sample size of 209 patients was deemed necessary to calculate the impact of allergic disease in school attendance and performance with a ± 6% precision and a 95% CI [29].

## Results

### Baseline characteristics of study patients and accompanying adults

Of the 125 recruited patients, 12 were unevaluable, resulting in a study population of 113 patients with a mean (SD) age of 10 (3.3) years and time of disease evolution of 4.2 (2.6) years. Table 1 summarizes the demographic, clinical, and treatment characteristics of study patients. The mean (SD) number of visits to healthcare centers in the previous 12 months was 3.8 (3.2). Of the 113 patients, 23%, 41.6%, and 35.4% were recruited during the first (October–December), second (January–March), and third (April–June) quarters of the school year, respectively. The 113 accompanying caregivers had a mean (SD) age of 41.1 (6.4) years and were mostly the patients’ mothers (Table 2). A flow diagram of study patients is included in Figure S1 (Supporting information file).

**Table 1:** Demographic, clinical, and treatment characteristics of study patients, *n* (%) *N* = 113

**Demographic characteristics**	
Age, years	
Children (5–11)	76 (67.3)
Adolescents (12–17)	37 (32.7)
Sex	
Male	75 (66.4)
Female	37 (33.6)
Studies	
Primary school	77 (68.1)
Secondary school	36 (31.9)
**Clinical characteristics**	
Asthma severity (GEMA)	
Intermittent	50 (44.2)
Mild persistent	28 (24.8)
Moderate persistent	32 (28.3)
Unknown	3 (2.7)
Asthma control	
Complete	32 (28.3)
Good	51 (45.1)
Partial	26 (23.0)
Bad	1 (0.9)
Unknown	3 (2.7)
Allergic comorbidities	
Rhinitis	98 (87.6)
Conjunctivitis	27 (23.9)
Atopic dermatitis	34 (30.1)
Food allergy	9 (8.0)
Sensitizations to aeroallergens	
* Dermatophagoides sp.*	113 (100)
Other mites	43 (38.1)
Pollens	33 (29.2)
* Alternaria alternata*	4 (3.5)
**Treatment characteristics**	
Asthma medication	
Short-acting beta-agonists	62 (54.9)
Inhaled corticosteroids	45 (39.8)
Long-acting beta-agonists	20 (17.7)
Leukotriene receptor antagonists	17 (15)
Oral corticosteroids	2 (1.8)

Abbreviations: *GEMA*, Guía Española del Manejo del Asthma (Spanish guideline on the management of asthma)

**Table 2:** Demographic characteristics of caregivers, *n* (%) *N* = 113

Sex	
Male	24 (21.2)
Female	86 (76.1)
Unknown	3 (2.7)
Relationship	
Parent	105 (92.9)
Legal tutor	1 (0.9)
Grandparent	1 (0.9)
Other	4 (3.5)
Unknown	2 (1.8)
Employment status	
Employed	79 (69.9)
Unemployed	27 (23.9)
Retired	2 (1.8)
Other	2 (1.8)
Unknown	3 (2.7)

### Impairment of school/work performance and daily activities

Allergic disease impacted the school performance and daily activities of patients, resulting in 52.2% and 45.1% prevalence of school and activity impairment, respectively (Table 3). Of the 113 children, 30 (26.5%) reported missing at least 1 day of school, and 54 (47.8%) reported classroom impairment during the previous 7 days. Likewise, working performance (evaluated in the 79 employed caregivers) and daily activities of the caregivers were impaired by children’s allergic disease, with 38.0% and 23.0% prevalence of work and activity impairment, respectively (Table 3).

**Table 3:** Patients’ school impairment, caregivers’ work impairment, and patients’ and caregivers’ routine activities impairment due to allergic disease

**Patients,** ***n*** **= 113**	
With time missed (absenteeism), *n (%)* *n* = 113	30 (26.5)
With classroom impairment (presenteeism), *n (%)* *n* = 113	54 (47.8)
Overall impairment (absenteeism plus presenteeism), *n (%)* *n* = 113	59 (52.2)
With activity impairment, *n (%)* *n* = 113	51 (45.1)
% of impairment, *mean (SD*)^b^	
% of classroom time missed (absenteeism), *n* = 30	16.1 (12.8)
% of classroom impairment (presenteeism), *n* = 54	36.5 (22.7)
% of overall impairment (absenteeism plus presenteeism), *n* = 59	37.6 (24.4)
% of activity impairment, *n* = 51	42.6 (25.6)
**Caregivers, *n* = 113**	
With time missed (absenteeism), *n (%)* *n* = 79^a^	24 (30.4)
With while working impairment (presenteeism), *n (%)* *n* = 79^a^	25 (31.6)
Overall impairment (absenteeism plus presenteeism), *n (%)* *n* = 79^a^	30 (38.0)
Caregivers with activity impairment, *n* (%) *n* = 113	26 (23.0)
% of impairment, *mean (SD)*^b^	
% of work time missed (absenteeism), *n* = 24	11.4 (9.4)
% of while working impairment (presenteeism), *n* = 25	40.8 (27.4)
% of overall impairment (absenteeism plus presenteeism), *n* = 30	38.9 (29.4)
% of activity impairment, *n* = 26	32.7 (23.2)

*SD*, standard deviation

^a^Number of employed caregivers

^b^Patients/caregivers with the corresponding impairments

### Effects of immunotherapy on school performance and asthma evolution

**Fig. 1:**
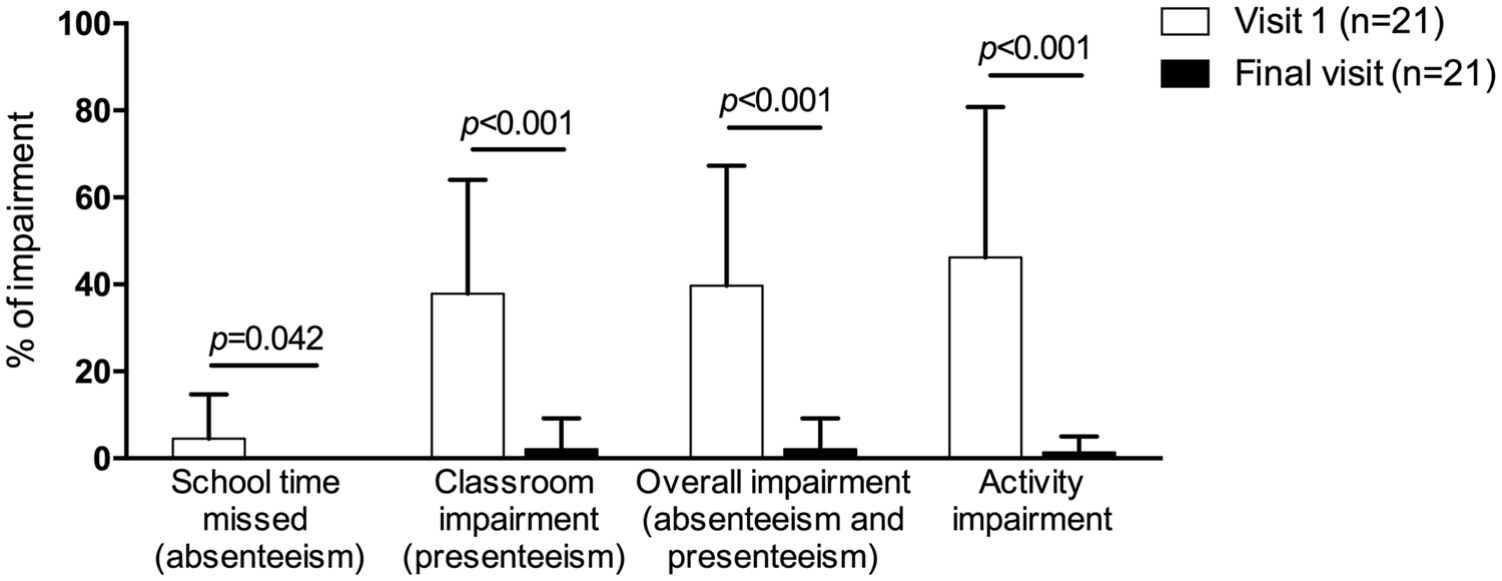
School time missed, classroom impairment, overall impairment, and activity impairment in patients who received allergen immunotherapy with a MicroCrystalline Tyrosine-associated house dust mite allergoid and attended the final visit at the indicated timepoints. Columns and error bars represent mean percentages and standard deviation, respectively. *p*-values were calculated using the Wilcoxon test

**Fig. 2:**
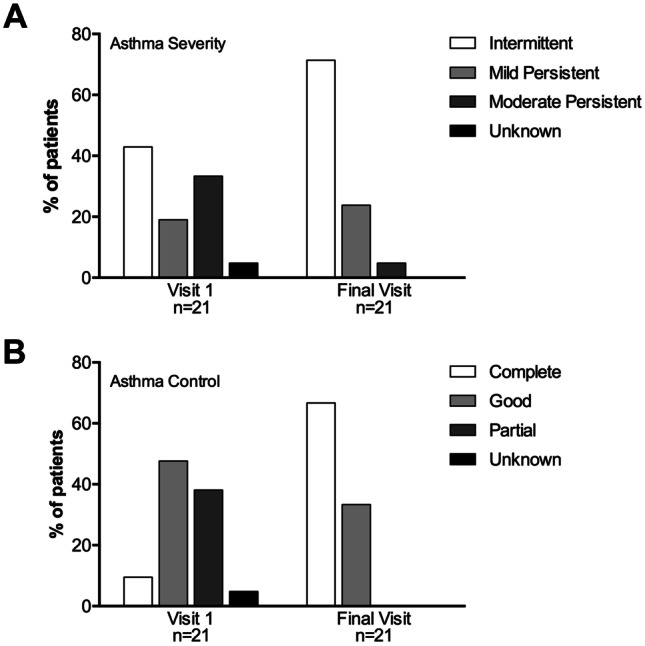
Asthma severity (**A**) and control (**B**) at visit 1 and after 1 year (final visit) of treatment with allergen immunotherapy with a microcrystalline tyrosine-adsorbed house dust mite allergoid. McNemar test, *p* = 0.021 (**A**) and *p* = 0.001 (**B**)

**Fig. 3:**
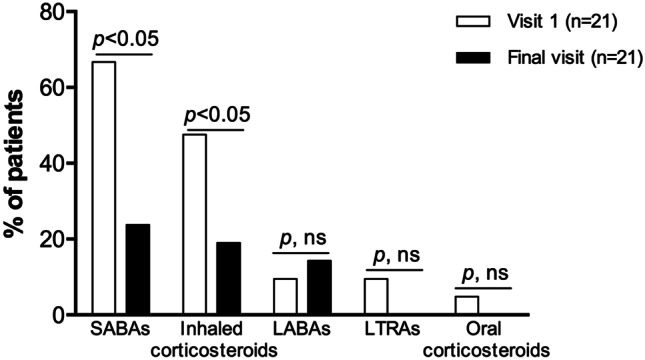
Use of medication to treat asthma before and after 1 year of treatment with allergen immunotherapy with a MicroCrystalline Tyrosine-associated house dust mite allergoid. LABAs, long-acting beta-2 agonists; LTRAs, leukotriene receptor antagonists; ns, not significant; SABAs, short-acting beta-2 agonists. *p*-values were calculated using the McNemar test

Of the 113 patients evaluated at V1, 65 (57.5%) reported school/activity impairment and were candidates to receive AIT. Of those, 24 were not prescribed AIT based on physicians’ criteria or refused AIT treatment for personal decision and 41 (63.1%) were prescribed and received AIT with Acarovac Plus® (Figure S1). Twenty patients did not attend school during the week before the final visit due to the lockdown during the COVID-19 pandemic, precluding the assessment of school performance. WPAI + CIQ:AS scores of the 21 (51.2%) patients who completed the first year of treatment and attended FV revealed significantly decreased absenteeism, presenteeism, overall school impairment, and activity impairment (Fig. 1). Asthma classification changed towards significantly decreased severity (*p* = 0.021) (Fig. 2A) and significantly improved control (*p* = 0.001) (Fig. 2B). Accordingly, the use of SABA and inhaled corticosteroids significantly decreased 1 year post-AIT (*p* < 0.05) (Fig. 3), and the mean (SD) number of unscheduled visits to the healthcare center decreased from 4.1 (2.8) to 1.9 (2.3) (*p* = 0.003).

## Discussion

Results from this study showed a considerable prevalence of asthma-related school and activity impairment in children and adolescents allergic to HDMs and work and activity impairment in their caregivers. School and work impairment resulted from both missed school/work time and classroom/at-work impairment, with a greater contribution of the latter. In asthmatic children receiving AIT, the severity of asthma decreased, and their control improved, with concomitantly decreased use of asthma medication, unscheduled visits to the healthcare center, and school and activity impairment 1 year after AIT.

The impact of allergic rhinitis in children’s school performance and activities and that of allergic asthma in work and activity of adult patients have been evaluated in previous studies [30–32]. However, despite its persistence throughout the school year, the impact of perennial allergic asthma in children’s and adolescents’ daily lives and in those of their caregivers remains to be fully investigated. This study focused on the impact of perennial asthma due to allergy to HDMs, the most common allergen in children’s homes during all seasons, in school performance. Using the WPAI + CIQ:AS, a standardized and widely used questionnaire specifically evaluating the impact of allergic disease, this study assessed the two dimensions of school performance: absenteeism and presenteeism [24].

The rate of school absenteeism due to asthma symptoms in this study (26.5%) lied within the rates previously reported in different countries worldwide (ranging 9.1 to 61%). [18, 19, 33]. Overall, this and previous studies were heterogeneous regarding time periods assessed (ranging from 7 days in this study to 12 months) and age groups, precluding direct comparisons among them. Other studies have reported increased missed school days in children with asthma compared to those without doctor-diagnosed asthma, but few of these studies tracked absences, and therefore, the direct relationship between missing school and asthma symptoms remained mostly unassessed [20, 22, 34]. Even though most of this study’s population had mild persistent and intermittent asthma and good/complete asthma control, asthma symptoms had a substantial impact on the patients’ school performance and daily activities. Despite differences in their design, this and previous studies revealed a relationship between asthma and school impairment, and potentially, decreased academic achievements [11, 35].

Even though several studies have reported an increased probability of poor academic performance in children with asthma [11, 34, 36], few have quantified presenteeism [36]. The WPAI + CIQ:AS allowed to assess the impact of asthma in school/work presenteeism (i.e., at-school/at-work productivity loss) and showed higher rates of presenteeism (47.8%) compared to those of absenteeism (26.5%), underscoring a significant contribution of presenteeism to overall school impairment.

Children’s asthma substantially impacts family life, including parents’ sleep time and activities, and increases missing workdays, which have been shown to generate indirect costs higher than those associated with the parent’s own illness [8, 9]. Likewise, the effects of asthma on work productivity have been reported to be mostly due to caregivers’ presenteeism (as opposed to absenteeism), also referred to as at-work productivity [1, 9]. In this context, the results from this study indicate that the impact of schooled patients’ asthma on the overall work performance of their caregivers (i.e., absenteeism and presenteeism) may contribute to increasing indirect asthma costs.

Children who had received AIT to treat perennial allergic asthma due to HDM showed a favorable disease evolution, resulting in significantly decreased asthma-related school impairment in those who attended the FV. Even though sample attrition was significant in this study, the marked reduction in school impairment was not unexpected, considering the previously reported association between the degree of asthma control and days of missed school—frequently overestimated by parents—and its severity [8, 11, 20]. In a previous study assessing the effects of this AIT in adults, work, study, and activity impairment due to rhinitis symptoms were 21.0%, 21.2%, and 22.0%, respectively, lower than the 37.6% and 42.6% for school and activity impairments, respectively, reported in this study [30]. Similar to this study, AIT was associated with increased academic and work productivity and decreased activity impairment [30]. Results from this study point to AIT with a MCT-adsorbed HDM allergoid as an effective therapeutic strategy to reduce school impairment by improving asthma evolution.

Results from this study should be interpreted in the context of limitations related to the study design and the sample used to evaluate AIT effects. Given its real-world nature, this study lacked a control arm, and consequently, changes in effectiveness variables, which were mostly subjective (i.e., symptoms and use of medication), may have been influenced by a placebo effect. However, asthma evolution was also measured using the number of visits to the healthcare center, which is a robust objective measure, and school impairment is an objective variable measured in the previous 7 days, and therefore, unlikely influenced by a potential placebo effect and recall bias. Regarding sample size, the number of patients available for analysis of the primary outcome (i.e., impact of allergic disease on school performance) did not reach the calculated study’s sample size, as an insufficient number of patients was recruited. Further sample attrition for the analysis of the effects of AIT was due to the number of patients eligible to continue in the study (i.e., those with impairments) and the criteria to prescribe AIT. Finally, school lockdown during the COVID-19 pandemics limited the number of patients with available WPAI + CIQ:AS at 1 year of follow-up. In addition, it is possible that those patients with worse disease evolution were lost-to-follow-up, resulting in selection bias. Nevertheless, the reduced sample size provided sufficient statistical power to detect absenteeism and presenteeism and statistically significant differences after only 1 year of AIT treatment. Future studies with prolonged follow-up periods should be designed to assess the long-term effects of this AIT. Lastly, the unique characteristics of the Spanish healthcare system, free and available for everyone, facilitate access to healthcare and prescription drugs, precluding the generalizability of the results to countries with non-universal healthcare systems. Despite its limitations, this study was able to capture the impact of HDM allergic asthma in children’s and their caregivers’ school/work and activity impairment and the effects of AIT in the population encountered in the real-world setting, without the strict selection criteria of clinical trials. AIT resulted in favorable disease evolution and decreased school/activity impairment, and, potentially, a favorable impact on children’s QoL. In addition, results from this study suggest that the indirect costs associated with loss of caregivers’ work productivity due to inappropriate disease management may be reduced by therapeutic strategies able to achieve symptom control.

In conclusion, both absenteeism and presenteeism contribute to the loss of school productivity and loss of activity/leisure time due to allergic asthma symptoms in children sensitized to HDMs. AIT with a modified and MCT-associated HDM extract is likely to be effective in improving asthma control and decreasing its severity, with a concomitantly decreased school and activity impairment. Furthermore, children’s asthma impacts work absenteeism, presenteeism, and daily activities of their caregivers, contributing to indirect costs of schooled children’s asthma.

## Supplementary Information

Below is the link to the electronic supplementary material.

Supplementary file1 (DOCX 5943 KB)

## Data Availability

The datasets used and/or analyzed during the current study are available from the corresponding author upon reasonable request.

## Abbreviations

AIT: Allergen immunotherapy
GEMA: “Guía Española del Manejo del Asma”
GINA: Global Initiative for Asthma
HDMs: House dust mites
LABAs: Long-acting beta-2 agonists
LTRAs: Leukotriene receptor antagonists
MCT: MicroCrystalline Tyrosine
QoL: Quality of life
SABAs: Short-acting beta-2 agonists
SD: Standard deviation
WPAI+CIQ: AS Work Productivity Impairment Questionnaire plus Classroom Impairment Questions: Allergy Specific
