# Supplementary material for: Impact of house dust mite-driven asthma on children’s school performance and activity
Source: Eur J Pediatr. 2021 Dec 21;181(4):1567–74. doi: 10.1007/s00431-021-04346-y (PMC8691866; doi:10.1007/s00431-021-04346-y)
Supplement: Supplementary file 1 — Supplementary file1 (DOCX 5943 KB) [file 431_2021_4346_MOESM1_ESM.docx]

**Supplementary Information for the Submission by Gómez et al. “Impact of House Dust Mite-Driven Asthma on Children's School Performance and Activity”.**

#
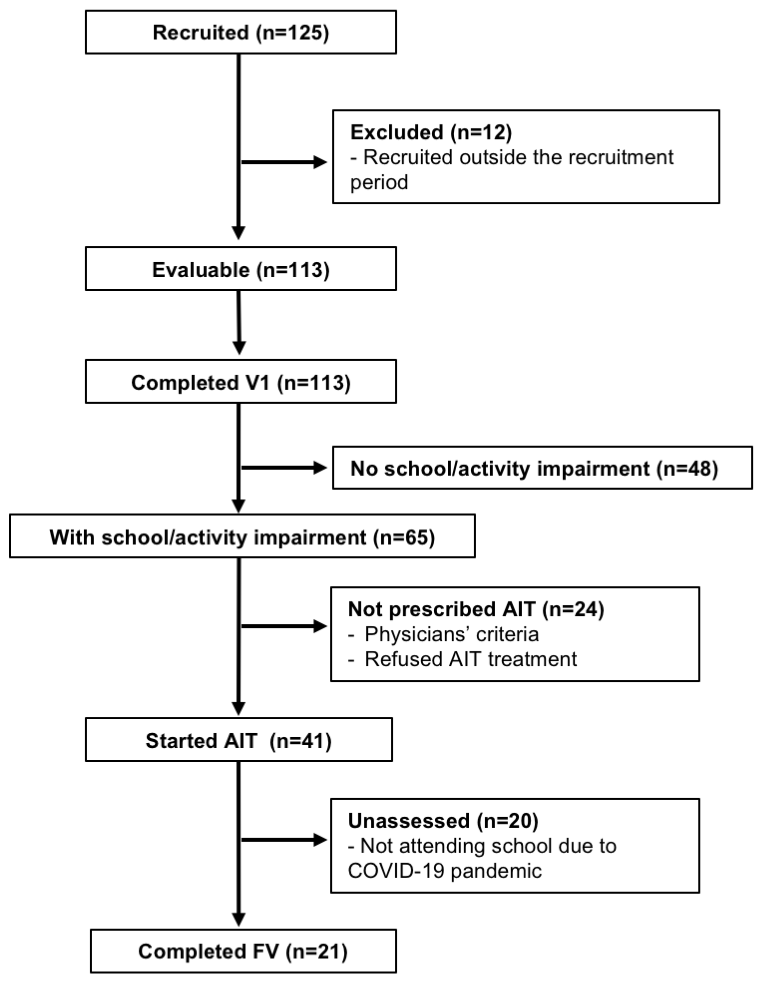
Supplementary Figure

**Figure S1.** Flow diagram of study patients. AIT, allergen immunotherapy; FV, final visit; V1, visit 1.
